# Conventional and two-dimensional strain echocardiography in predicting postoperative atrial fibrillation after coronary artery bypass grafting surgery

**DOI:** 10.22088/cjim.14.1.60

**Published:** 2023

**Authors:** Seyed Arash Bagherinejad Somesarayi, Leili Faridi, Kamran Mohammadi, Babak Kazemi Arbat, Mehran Rahimi, Razieh Parizad, Mehrnoush Toufan Tabrizi

**Affiliations:** 1 Cardiovascular Research Center, Tabriz University of Medical Sciences, Tabriz, Iran

**Keywords:** Conventional echocardiography, LA 2D-strain echocardiography, Atrial fibrillation, Coronary artery bypass grafting

## Abstract

**Background:**

Atrial fibrillation (AF) is a common complication after heart surgeries. Advances in imaging technologies and an understanding of the pathophysiology of preoperative left atrial (LA) dysfunction can lead to more definitive potential therapeutic approaches. This study aimed to determine the role of conventional echocardiography and LA two-dimensional (2D) strain echocardiography in assessing LA function and predicting POAF after CABG surgery.

**Methods:**

All patients with sinus rhythm who underwent CABG surgery were enrolled. All the patients had undergone conventional echocardiography and LA 2D-strain echocardiography 24 hours before surgery. In addition to demographic, clinical, and perioperative features, electrocardiogram (ECG) and Holter monitoring were recorded.

**Results:**

Of the 105 patients included, 85 patients (81%) were men with a mean age of 60.26±10.61 years. POAF was seen in 22.9% of patients during hospitalization, and AF duration was 10 hours (median; IQR: 2.0-19.5). AF patients had a higher LA volume index (LAVI) than patients with sinus rhythm (p=0.018). Patients with sinus rhythm had higher rates of LA reservoir (26.97±6.87 VS. 20.46±4.27, p<0.001), LA contractile (14.98±3.68 VS. 12.76±3.72, p =0.012) and LA global strain (24.28±6.57 VS. 17.71±4.11, p<0.001) than AF patients. The results of the multivariate logistic regression showed that LAVI (p=0.014) and LA global strain (p=0.027) were independent predictors of AF detection.

**Conclusion:**

Compared to conventional echocardiography, 2D-strain echocardiography is a more effective diagnostic method to predict the possibility of post-CABG AF.

As the most common complication after cardiac surgeries, postoperative atrial fibrillation (POAF) is seen in 20-50% of the patients and involves serious complications such as stroke, renal dysfunction, and death (1). POAF also places a critical financial burden on healthcare systems through prolonged hospitalizations and increased costs (1, 2). Patients undergoing CABG and valvular surgery have the highest risk of developing POAF, ranging from 60% to 80%(3). POAF usually converts to sinus rhythm without intervention(4). Recent data suggest that, compared to non-valvular AF, POAF has a similar long-term thromboembolic profile with increased mortality (5). The clinical predictors of POAF are essential in identifying patients at risk for POAF. LA dysfunction may significantly determine patients at risk for postoperative atrial fibrillation (POAF) after CABG surgery (6, 7).

Advances in imaging techniques and an understanding of AF pathophysiology can lead to more definitive potential therapeutic approaches. According to recent reports, two-dimensional (2D), speckle-tracking strain imaging is a practical and reproducible method to evaluate LA function by assessing LA deformation dynamics, a new, angle-independent method for quantitative measurement of LA contraction and myocardial passive deformation (8, 9). Several studies have reported a significantly lower LA global strain in patients who develop POAF. Increased age is the most important risk factor of POAF as aging decreases LA reservoir function (LASs, LASRs) and contractile function (LASRa), and increases atrial fibrosis result from the aging process (10).

This study aimed to determine the role of and compare conventional echocardiography and LA two-dimensional (2D) strain echocardiography in assessing LA function and predicting POAF after CABG surgery.

## Methods

This study was approved by the medical ethics committee at the Tabriz University of Medical Sciences, Tabriz, Iran. Exclusion criteria for the present study were: (1) patients with no written consent to participate in the study, (2) patients diagnosed with acute coronary syndromes (ACS) who underwent emergent CABG surgery, (3) lack of suitable image for 2D-strain echocardiography, (4) any kind of rhythm except normal sinus rhythm, (5) thyroid dysfunction or being treated for thyroid diseases, (6) any electrolyte abnormality, (7) being treated with antiarrhythmic drugs (except beta-blockers), and (8) existence of more than mild valvular problem.

In this study, 105 patients with sinus rhythm undergoing elective CABG surgery at the Shahid Madani Hospital, Tabriz, Iran, from August 2020 to February 2021 were included. All the patients had undergone conventional echocardiography and LA 2D-strain echocardiography 24 hours before surgery. ECG was taken, p wave interval was measured, and echocardiographic information was recorded. Before surgery, all ECGs were taken with a single machine (Medical ECONET, CARDIO M 12 - Channel Resting ECG). Demographic information was also recorded in the checklist, including age, sex, risk factors (hypertension or taking antihypertensive drugs, self-reported diabetes or taking anti-diabetic drugs), obesity (Body Mass Index > 30), and history of AF in first-degree relatives, and angiographic information including the number and name of vessels involved. Then, the patients’ surgical information, including the number of received grafts, pump time, cross-clamp time, intraoperative complications (such as arrhythmia), off- or on-pump surgery, were added to the checklist. Also, the types of drugs received by the patients were completely included in the checklist. The patients underwent ECG Holter monitoring in the ICU for 72 hours from the time of arrival to ICU to determine the incidence of POAF. 

A cardiac electrophysiologist analyzed the Holter data. Rhythm disturbance (AF) was confirmed on ECG and Holter monitoring. The detection of AF with irregular rhythm lasted for more than 30 seconds was established by variable R-R interval and absence of p-wave. The diagnoses were made by two specialists who were blind to the patients and their data. All of the echocardiographic findings were reported according to the American Society of Echocardiography guidelines (11). Echocardiography was performed using a commercially available Philips EPIQ 7 Cardiology Ultrasound Machine. LVEDD and LVESD were measured in parasternal long-axis view, and LVEDV, LVESV, and LVEF were measured by the modified Simpson’s method.

Diastolic function was assessed by mitral inflow velocity (deceleration time, E/A, A wave, E wave). Tissue Doppler was measured by placing a pulsed wave sample volume in the medial and lateral corner of the mitral annulus in the apical four-chamber view. LA volume was calculated using the biplane method of disks, and LA volume index was obtained based on body surface area.

All strain parameters were measured by speckle-tracking imaging in apical four-chamber view and apical two-chamber view with a frame rate of 60-80 frames/sec. After manually tracking the LA endocardial border, the software automatically tracked the myocardium. We reviewed all software evaluations to ensure that LA motion tracking was accurate and adequate, and strain results were obtained in two views. LA strain diagrams were created automatically by the relevant software. 

**Figure 1:**
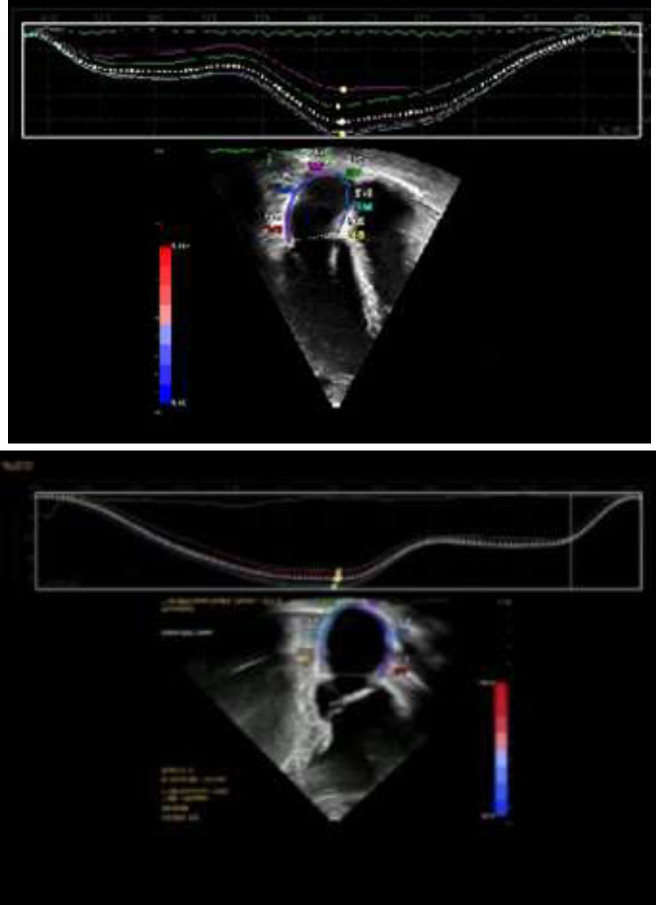
LA strain curve: Two‐dimensional speckle tracking strain demonstrating phasic LA strain (LASr, LAScd, and LASct) from the apical 4 chamber view and apical 2 chamber view. Segmental strain from 6 segments as well as LA global strain (white dotted line) is shown

The reference point for image analysis was the appearance of the QRS complex. The two peaks, “reservoir” and “contractile”, were assessed in the strain diagram. We divided the LA wall into basal, mid, and apical segments in the apical 4- and 2-chamber views. LA reservoir strain (LASr), measured in the reservoir phase, is the difference between the onset of filling and end-diastole. LA conduit strain (LAScd) is the difference between the onset of atrial contraction and the onset of filling. LA contractile strain (LASct), observed corresponding to atrial systole, was measured as the difference between end-diastole and onset of the atrial filling (Figure1) (12).

**Statistical Analysis: **Data were presented as mean (±standard deviation) and frequency (percentage) for quantitative and qualitative variables. Data analysis was performed in SPSS software version 22.

The normality of data distribution was assessed using the Kolmogorov–Smirnov test. Based on the type of skewness, a suitable conversion was carried out for variables without a normal distribution to normalize data distribution. Chi-square test was used to compare the qualitative variables of the participants between the two groups, and independent samples t-test or ANOVA was used to compare their quantitative variables. To control for the intervening variables and the basic measurements of the variables, an analysis of covariance (ANCOVA) was used. Given the multi-factorial nature of AF, univariate and multivariate methods were used to analyze the data and the effect of the strain. In this study, P values less than 0.05 were considered statistically significant.

## Results

The results of this study illustrated that most of the patients (N=85, 81%) were men, and the overall mean age was 60.26 ± 10.61 years. The clinical characteristics and underlying variables of echocardiography are presented in Table 1. Fifty percent of the patients underwent off-pump CABG surgery, 50% underwent on-pump surgery with 98.52±22.44 minutes pump time. All of the patients received beta-blockers, aspirin, and statins before surgery but no antiarrhythmic medication. All the patients in this study were discharged except 5 (4.8%) patients who had a stroke and passed away. POAF was seen in 22.9% of patients during hospitalization, and AF's duration was 10 hours (median; IQR: 2.0-19.5 hours). Of the 24 patients who developed AF rhythm, 6 (25%) patients spontaneously converted to sinus rhythm without medication, while 18 (75%) patients converted to sinus rhythm after receiving Amiodarone.

The AF patients were older with a mean age of 64.04±9.21 (p=0.039). The average number of hospitalization days (15.41± 4.88 vs. 17.58±6.11, p=0.155) and ICU stay (5.43±2.64 vs. 8.95 ± 6.84, p = 0.002) were higher in the AF group. This difference, however, was only significant in the average length of ICU hospitalization between the two groups (p=0.002). The mean pump time in the AF patients was 14.48 minutes longer than the patients with sinus rhythm, which was statistically insignificant (P=0.523). AF occurred in all the patients within 72 hours after surgery. 

Sex and other risk factors (hypertension, diabetes, obesity, history of chemotherapy, and family history of AF) were not significantly different between the two groups. The mean P wave duration was higher in AF patients, but there was no significant difference between the inferior leads.

AF patients had higher LAVI than those in the sinus rhythm group (P=0.018). No significant difference was found between the two groups regarding LVEDD, LVESD, LVEDV, LVEF, E/e, E/A, and DT variables. Patients with sinus rhythm had higher rates of LA reservoir (26.97±6.87 VS. 20.46 ± 4.27, p <0.001), LA contractile (14.98 ± 3.68 VS. 12.76 ±3.72, p=0.012) and LA global strain (24.28 ± 6.57 VS. 17.71±4.11, p<0.001) than AF patients (Table 2).

The results of the multivariate logistic regression showed that LAVI (P=0.014) and LA global strain (P=0.027) were independent predictors of AF detection (Table 3). Consequently, the probability of developing AF increases with an increase in LAVI and decreases with a decrease in LA global strain.

**Table 1:** Clinical and echocardiographic characteristics

**Variables**	**Total**	**No AF(N=81)**	**AF(N=24)**	**P-value**
Male sex, No (%)	85(81)	66(81.5)	19(79.2)	0.503
Age, mean±SD	60.26±10.61	59.14±10.79	64.04±9.21	0.039*
HTN, No (%)	74(70.5)	55(67.9)	19(79.2)	0.212
DM, No (%)	33(31.4)	23(28.4)	10(41.7)	0.163
BMI>30, No (%)	1(1)	1(1.2)	0	0.771
Bio-chemo therapy, No (%)	0	0	0	---
AF history, No (%)	0	0	0	---
Smoking, No (%)	42(40)	33(40.7)	9(37.5)	0.485
Angiography, No (%)				
LML>50%,	2(1.9)	1(1.3)	1(4.2)	0.310
1VD	6(5.8)	5(6.3)	1(4.2)	
2VD	26(25)	19(23.8)	7(29.2)	
3VD	64(61.5)	52(65)	12(50)	
LM+1VD	1(1)	1(1.2)	0	
LM+2VD	1(1)	0	1(4.2)	
LM+3VD	4(3.8)	2(2.5)	2(8.3)	
**ECG**				
P wave duration II (second)	0.06±0.01	0.06±0.01	0.07±0.01	0.002*
P wave duration III (second)	0.04±0.01	0.04±0.01	0.05±0.01	<0.001*
P wave duration AVF(second)	0.04±0.01	0.04±0.01	0.05±0.01	0.003*
**Infarcts**				
V1-V4, No (%)	1(1)	1(1.2)	0	0.605
V1-V6, No (%)	13(12.4)	11(13.6)	2(8.3)	
V1-V6+1, AVL, No (%)	3(2.9)	2(2.5)	1(4.2)	
**Inferior**	9(8.6)	5(6.2)	4(16.7)	
INF+RV, No (%)	1(1)	1(1.2)	0	
NO Infarcts, No (%)	78(74.3)	61(75.3)	17(70.8)	
**Treatments**				
Aspirin, No (%)	105(100)	77(95.1)	22(91.7)	0.618
Beta blocker, No (%)	105(100)	75(92.6)	21(87.5)	0.424
Calcium blocker, No (%)	27(25.7)	18(22.2)	9(37.5)	0.183
TNG, No (%)	5(4.8)	3(3.7)	2(8.3)	0.321
Nitrate, No (%)	58(55.2)	45(55.6)	13(54.2)	0.999
Diuretic, No (%)	77(73.3)	60(74.1)	17(70.8)	0.795
Statin, No (%)	105(100)	76(93.8)	22(91.7)	0.658
Clopidogrel, No (%)	79(75.2)	60(74.1)	19(79.2)	0.789
Amiodarone, No (%)	4(3.8)	0	4(16.7)	0.002
Lidocaine, No (%)	0	0	0	---
Anti-Arrhythmia, No (%)	0	0	0	---
**LAD **				
Proximal, No (%)	70(66.7)	55(67.9)	15(62.5)	0.630
MID, No (%)	56(53.3)	43(53.1)	13(54.2)	0.999
Distal, No (%)	12(11.4)	11(13.6)	1(4.2)	0.289
**LCX **				
Proximal, No (%)	42(40)	32(39.5)	10(41.7)	0.999
MID, No (%)	0	0	0	---
Distal, No (%)	23(21.9)	19(23.5)	4(16.7)	0.583
**RCA **				
Proximal, No (%)	29(27.6)	19(23.5)	10(41.7)	0.117
MID, No (%)	34(32.4)	26(32.1)	8(33.3)	0.999
Distal, No (%)	27(25.7)	19(23.5)	8(33.3)	0.425
Diag, No (%)	38(36.2)	29(35.8)	9(37.5)	0.879
OM1, No (%)	41(39)	31(38.3)	10(41.7)	0.814
PDA, No (%)	15(14.3)	13(16)	2(8.3)	0.511
**Graft **				
1, No (%)	13(12.5)	11(13.8)	2(8.3)	0.293
2, No (%)	37(35.6)	25(31.3)	12(50)	
3, No (%)	48(46.2)	40(50)	8(33.3)	
4, No (%)	6(5.8)	4(5.)	2(8.3)	
**Cardio pulmonary**				
On pump, No (%)	51(50)	40(51.3)	11(45.8)	0.641
Off pump, No (%)	51(50)	38(48.7)	13(54.2)	
Pump time (minutes), mean±SD	98.52±22.44	91.42±20.51	105.9±30.02	0.523*
**Intraoperative complications**				
Arrhythmia, No (%)	0	0	0	---
**Pack Cell**				
No, No (%)	41(39)	35(43.2)	6(25)	0.057
1, No (%)	45(42.9)	35(43.2)	10(41.7)	
2, No (%)	14(13.3)	7(8.6)	7(29.2)	
3, No (%)	3(2.9)	3(3.7)	0	
4, No (%)	2(1.9)	1(1.2)	1(4.2)	
Hospitalization (day), mean±SD	15.91±5.23	15.41±4.88	17.58±6.11	0.155*
ICU (day), mean±SD	6.23±4.23	5.43±2.64	8.95±684	0.002*
**Status**				
Dead, No (%)	5(4.8)	3(3.7)	2(8.3)	0.321
Alive, No (%)	100(95.2)	78(96.3)	22(91.7)	

*: Mann – whiteny U, AF: Atrial Fibrillation, HTN: Hypertension, DM: Diabetes Mellitus, BMI: Body mass index, 1VD: One vessels Disease, 2VD: Two vessels Disease, 3VD: Three vessels Disease, INF+RV: Inferior and Right Ventricular, TNG: Nitroglycerin, LM: Left main, RCA: Right coronary artery, LCX: Left circumflex artery, PDA: Patent ductus arteriosus

**Table 2:** Echocardiography characteristics

**Variables**	**Total**	**No AF(N=81)**	**AF(N=24)**	**P-value**
**Echocardiography Conventional**				
LVEDD mm	46.01±5.45	46.09±5.41	45.75±5.74	0.785**
LVESD mm	33.47±6.41	33.34±6.18	33.91±7.25	0.704**
LVESV cc	50.31±23.99	50.05±21.09	51.16±32.48	0.717*
LVEDV cc	94.63±27.81	96.46±29.10	88.44±22.35	0.395*
LVEF %	48.53±9.52	49.08±10	46.71±7.58	0.345*
LAVI m1/m2	30.13±8.01	29.15±7.46	33.61±9.03	0.018**
E/e	8.94±2.34	8.96±2.33	8.87±2.46	0.844*
E/A	0.87±0.38	0.89±0.41	0.83±0.24	0.997*
DT mse	189.82±42.62	188.06±42.73	196.04±42.57	0.431**
**Echocardiography strain**				
LA reservior strain %	25.57±6.89	26.97±6.87	20.46±4.27	<0.001**
LA contractile strain %	14.48±3.78	14.98±3.68	12.76±3.72	0.012**
LA global strain %	22.83±6.68	24.28±6.57	17.71±4.11	<0.001**

LVEDD: Left ventricular end-diastolic diameter, LVESD: Left ventricular end-systolic diameter, LVESV: Left ventricular end systolic volume, LVEDV: Left ventricular end diastolic volume, LVEF: Left ventricular ejection fraction, LAVI: Left atrial volume index, E/e: Early filling/early diastolic, E/A: E wave / the A wave, DT: Deceleration time, LA: Left atrial, *: Mann – whitney U **: Independent samples T-test

**Table 3:** The result of logistic regression analysis

**Variables**	**Univariate**		**Multivariate**	
	**OR(95%CI)**	**P-value**	**OR(95%CI)**	**P-value**
Conventional				
LVEDD mm	0.98(0.91-1.07)	0.782		
LVESD mm	1.01(0.94-1.08)	0.700		
LVESV cc	1.01(0.98-1.02)	0.842		
LVEDV cc	0.98(0.97-1.01)	0.217		
LVEF %	0.97(0.92-1.02)	0.282		
LAVI m1/m2	1.07(1.01-1.13)	0.023	1.08(1.01-1.16)	0.014
E/e	0.98(0.81-1.19)	0.881		
E/A	0.63(0.15-2.64)	0.530		
DT mse	1.01(0.99-1.01)	0.427		
Strain				
LA reservoir strain %	0.85(0.78-0.93)	<0.001	0.9(0.75-1.07)	0.249
LA contractile strain %	0.85(0.75-0.97)	0.016	1.23(0.97-1.57)	0.08
LA global strain %	0.83(0.75-0.91)	<0.001	0.8(0.65-0.97)	0.027

LVEDD: Left ventricular end-diastolic diameter, LVESD: Left ventricular end-systolic diameter, LVESV: Left ventricular end systolic volume, LVEDV: Left ventricular end diastolic volume, LVEF: Left ventricular ejection fraction, LAVI: Left atrial volume index, E/e: Early filling/early diastolic, E/A: E wave / the A wave, DT: Deceleration time

## Discussion

As one of the most common complications of CABG surgery, atrial fibrillation requires special attention to achieve early diagnosis, preventive treatments, and timely interventions. In addition to monitoring patients in the ICU to perform emergency interventions, other predictive diagnostic modalities that can be helpful in the long term and during patient discharge to the ward and reduce the need for 24-hour care are critical in cardiac surgeries, especially CABG (1). In this study, two modalities, namely 2D-strain and conventional echocardiography, were evaluated in terms of their ability to predict atrial fibrillation. The present study demonstrated that most patients were male. Overall, the prevalence of the CABG is higher in men, ranging from 57.0% to 99.0%, and the mean age ranges from 57 years to 76 years (13, 14). Aging causes a decrease in the premature diastolic filling rhythm (5).With deceleration of early diastolic filling, late atrial contraction increases in compensation and leads to LA enlargement. As a result of aging and LA dysfunction, POAF may become more common in older patients (4, 5, 15). 

In our study, the mean on-pump time was 98.52 ± 22.44 minutes which did not differ significantly between the two groups. In a meta-analysis including 16,261 patients, it was demonstrated that off-pump CABG leads to a lower incidence of POAF but not to any reduction in myocardial infarction or mortality within the first 30 days after the surgery (16). In contrast, another meta-analysis enrolling 10,954 participants concluded there is no statistically significant difference in terms of major adverse cardiovascular (and cerebrovascular) events (MACCE) between on- and off-pump CABG (17).

The prevalence of AF rhythm in this study was 22.9% (24 patients). AF has been reported in 5–40% of patients in the early postoperative period CABG surgery (18, 19). The average incidence of new-onset AF in post-CABG patients is 25.5% (20). In our study, the mean P wave duration with standard 12-lead ECG was 70±10 ms in lead II in AF patients (significantly higher than patients with sinus rhythm). In a prospective study conducted in China, preoperative P wave duration was an independent predictor of POAF, and preoperative P wave durations of more than 105ms were reported to have the best predictive value (sensitivity of 74%, specificity 65%). Meta-analysis was also performed, and preoperative P wave duration was significantly higher in AF patients with a weighted mean difference of 3.95ms (21). In this study, we did not find an association between POAF and significant risk factors (HTN, DM, obesity, and smoking) and standard and tissue Doppler echocardiography (E/e', DT, E/A, LVEF). 

Recent studies have shown that LA strain becomes impaired in patients with paroxysmal AF before LA enlargement develops (22). LA remodeling can be confirmed by the amount of elasticity that indicates the amount of local myocardial deformity (23). Previous studies have shown the predictive value of LA strain in POAF. Electrical remodeling-related LA dysfunction may increase recurrent tachyarrhythmias, and this can be confirmed by showing the association between LA dysfunction using strain echocardiography and POAF development (22, 24). LA global strain and LAVI have been reported as independent predictors of post-CABG AF (25). The multivariate logistic regression in this study revealed that LAVI and LA global strain were independent predictors of POAF. An increase in LAVI and a decrease in LA global strain is significantly associated with increased POAF after CABG surgery (26, 27). LA global strain demonstrates passive stretching of LA during LV systole and accurately measures LA reservoir function (28). Not surprisingly, patients with new–onset AF have been reported to have significantly worse LA reservoir strain and contractility strain than those without AF (10). 

The present study suffers from several limitations as follows. First, because of the relatively small sample size, the lack of long-term follow-up data, and the impossibility of performing a multivariate analysis commensurate with the risk factors, there is a need for prospective and long-term studies to confirm the results. The use of more advanced echocardiographic systems and the involvement of artificial intelligence software to analyze LA traction can help better predict the AF incidence. Since we had Holter monitoring for 72 hours in the ICU and subsequent ECGs were recorded daily or with symptoms, we cannot exclude missing short periods of asymptomatic POAF and hence may have underestimated the total burden of POAF. In the next step, not studying and not eliminating the effects of confounding, moderating, and controlling variables such as cardiac enzyme levels, BNP, inflammatory markers, social factors affecting cardiovascular diseases before, during, and after surgery that can affect the outcome of surgery, was another limitation of the present study.

This study found that 2D-strain echocardiography, compared to conventional echocardiography, is a more effective diagnostic tool in predicting the possibility of post-CABG AF. There is a need for large-scale prospective studies, which control all of the potential confounding factors to confirm the results. 

## References

[1] Hsu JC, Huang CY, Chuang SL (2021). Long term outcome of postoperative atrial fibrillation after cardiac surgery-a propensity score-matched cohort analysis. Front Cardiovasc Med.

[2] Chelazzi C, Villa G, De Gaudio AR (2011). Postoperative atrial fibrillation. ISRN Cardiol.

[3] Saxena A, Dinh DT, Smith JA (2012). Usefulness of postoperative atrial fibrillation as an independent predictor for worse early and late outcomes after isolated coronary artery bypass grafting (multicenter Australian study of 19,497 patients). Am J Cardiol.

[4] Aranki SF, Shaw DP, Adams DH (1996). Predictors of atrial fibrillation after coronary artery surgery: current trends and impact on hospital resources. Circulation.

[5] Gabrielli L, Corbalan R, Córdova S (2011). Left atrial dysfunction is a predictor of postcoronary artery bypass atrial fibrillation: association of left atrial strain and strain rate assessed by speckle tracking. Echocardiography.

[6] Mandegar M, Marzban M, Lebaschi A (2008). Cardiovascular risk factors and in-hospital mortality in 1258 cases of coronary artery bypass surgery in Tehran heart center. Acta Medica Iranica.

[7] Ostovan MA, Darvish N, Askarian M (2014). The prevalence of risk factors of coronary artery disease in the patients who underwent coronary artery bypass graft, Shiraz, Iran: Suggesting a model. Int Cardiovasc Res J.

[8] Kislitsina ON, Cox JL, Shah SJ (2020). Preoperative left atrial strain abnormalities are associated with the development of postoperative atrial fibrillation following isolated coronary artery bypass surgery. J Thorac Cardiovasc Surg.

[9] Deferm S, Bertrand PB, Churchill TW (2021). Left atrial mechanics assessed early during hospitalization for cryptogenic stroke are associated with occult atrial fibrillation: A speckle-tracking strain echocardiography study. J Am Soc Echocardiogr.

[10] Verdejo HE, Becerra E, Zalaquet R (2016). Atrial function assessed by speckle tracking echocardiography is a good predictor of postoperative atrial fibrillation in elderly patients. Echocardiography.

[11] Mitchell C, Rahko PS, Blauwet LA (2019). Guidelines for performing a comprehensive transthoracic echocardiographic examination in adults: recommendations from the American Society of echocardiography. J Am Soc Echocardiogr.

[12] Fan JL, Su B, Zhao X (2020). Correlation of left atrial strain with left ventricular end-diastolic pressure in patients with normal left ventricular ejection fraction. Int J Cardiovasc Imaging.

[13] Shawon MSR, Odutola M, Falster MO, Jorm LR (2021). Patient and hospital factors associated with 30-day readmissions after coronary artery bypass graft (CABG) surgery: a systematic review and meta-analysis. J Cardiothorac Surg.

[14] Gaudino M, Hameed I, Farkouh ME (2020). Overall and cause-specific mortality in randomized clinical trials comparing percutaneous interventions with coronary bypass surgery: a meta-analysis. JAMA Intern Med.

[15] Rahimi M, Taban-Sadeghi M, Nikniaz L, Pashazadeh F (2021). The relationship between preoperative serum vitamin D deficiency and postoperative atrial fibrillation: A systematic review and meta-analysis. J Cardiovasc Thorac Res.

[16] Dieberg G, Smart NA, King N (2016). On- vs off-pump coronary artery bypass grafting: A systematic review and meta-analysis. Int J Cardiol.

[17] Takagi H, Watanabe T, Mizuno Y, Kawai N, Umemoto T (2014). A meta-analysis of large randomized trials for mid-term major cardio- and cerebrovascular events following off-pump versus on-pump coronary artery bypass grafting. Interact Cardiovasc Thorac Surg.

[18] Maisel WH, Rawn JD, Stevenson WG (2001). Atrial fibrillation after cardiac surgery. Ann Intern Med.

[19] Mariscalco G, Klersy C, Zanobini M (2008). Atrial fibrillation after isolated coronary surgery affects late survival. Circulation.

[20] Kerwin M, Saado J, Pan J (2020). New-onset atrial fibrillation and outcomes following isolated coronary artery bypass surgery: A systematic review and meta-analysis. Clin Cardiol.

[21] Wu F, Wu Y, Tao W, Zhao H, Shen D (2018). Preoperative P-wave duration as a predictor of atrial fibrillation after coronary artery bypass grafting: A prospective cohort study with meta-analysis. Int J Nurs Sci.

[22] Parwani AS, Morris DA, Blaschke F (2017). Left atrial strain predicts recurrence of atrial arrhythmias after catheter ablation of persistent atrial fibrillation. Open Heart.

[23] Kawakami H, Ramkumar S, Nolan M (2019). Left atrial mechanical dispersion assessed by strain echocardiography as an independent predictor of new-onset atrial fibrillation: a case-control study. J Am Soc Echocardiogr.

[24] Gu J Postoperative new-onset atrial fibrillation following cardiac surgery with special reference to potential new predictors. Aalborg Universitet 2016.

[25] Di Gioia G, Mega S, Nenna A (2017). Should pre-operative left atrial volume receive more consideration in patients with degenerative mitral valve disease undergoing mitral valve surgery?. Int J Cardiol.

[26] Her AY, Kim JY, Kim YH (2013). Left atrial strain assessed by speckle tracking imaging is related to new-onset atrial fibrillation after coronary artery bypass grafting. Can J Cardiol.

[27] Toufan M, Kazemi B, Molazadeh N (2017). The significance of the left atrial volume index in prediction of atrial fibrillation recurrence after electrical cardioversion. J Cardiovasc Thorac Res.

[28] Mondillo S, Cameli M, Caputo ML (2011). Early detection of left atrial strain abnormalities by speckle-tracking in hypertensive and diabetic patients with normal left atrial size. J Am Soc Echocardiogr.

